# *N*-Methyl-4-hydrazino-7-nitrobenzofurazan: a fluorogenic substrate for peroxidase-like DNAzyme, and its potential application

**DOI:** 10.1007/s00216-014-8119-7

**Published:** 2014-09-12

**Authors:** Joanna Kosman, Yu-Tang Wu, Agata Gluszynska, Bernard Juskowiak

**Affiliations:** Faculty of Chemistry, Adam Mickiewicz University in Poznan, Umultowska 89b, 61-614 Poznan, Poland

**Keywords:** Bioanalytical methods, Fluorescence, Nucleic acids, Molecular beacon, DNAzyme

## Abstract

Characterization and optimization studies of *N*-methyl-4-hydrazino-7-nitrobenzofurazan (MNBDH) as a new fluorogenic substrate in the peroxidation reaction catalyzed by DNAzyme are reported. The effects of pH, H_2_O_2_ concentration, metal-cation type, and the concentration and type of surfactant on the fluorescence intensity were investigated. The optimized reaction was subsequently used for the development of an assay for DNA detection based on a molecular-beacon probe. The use of a fluorogenic substrate enabled the detection of a single-stranded DNA target with a 1 nmol L^−1^ detection limit.

**Graphical Abstract:**
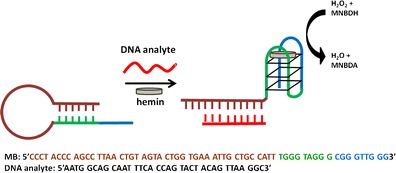
ᅟ

ᅟ

## Introduction

DNA is one of the most important molecules in living organisms. In addition to its biological function of storing genetic information, these macromolecules have proved to also be a useful material for nanotechnology [1], medicine [2], biotechnology [3], and the development of sensing devices [4]. One of the DNA systems that have attracted great attention is DNAzyme, which has peroxidase-mimicking activity [5]. To have catalytic activity, a DNA oligonucleotide must first adopt a G-quadruplex structure and then form a complex with a molecule of hemin. The peroxidase-mimicking DNAzyme catalyzes the reaction between hydrogen peroxide and an organic substrate. Through the selection of a proper substrate, this system can be successfully incorporated into new bioassays. Most of the developed assays are based on chemiluminescence with luminol as a substrate or on a colorimetric approach with 2,2ʹ-azino-bis(3-ethylbenzthiazoline)-6-sulfonic acid (ABTS) as a substrate [6–12]. Using these substrates, several analytical systems have been designed for the detection of analytes including metal ions [6, 7], DNA sequences [8, 9], proteins [10], enzymes [11], and others [12, 13].

Compared with protein enzymes, DNAzymes have several advantages that enable the development of simpler bioassays. First, they are thermally stable, do not require strict refrigerator storage, and can be heated even to 90 °C and cooled to room temperature with no effect on their catalytic activity. Second, DNA oligonucleotides are simple and cheap regarding synthesis, multiplication (by PCR), and purification. One of the most important advantages of deoxyribozymes, however, is their ability to hybridize. This makes it possible to design systems with selected features. A good example of such a system is a molecular-beacon probe (MB). Xiao et al. reported a system for DNA-target detection based on a molecular-beacon probe, which, after DNA-target hybridization to the MB loop, formed a peroxidase-mimicking DNAzyme that catalyzed ABTS oxidation [14]. This system enabled DNA detection at a 0.2 μmol L^−1^ concentration.

Enhancement of the analytical signal through DNAzyme action and use of a colorimetric detection technique enables the determination of analytes in the submicromolar concentration range. However, medicine and molecular biology are still seeking detection systems that enable the detection of molecules at nano or even picomolar levels. One solution that provides higher sensitivity is the use of fluorogenic substrates, which emit fluorescence after oxidation by a DNAzyme. Two fluorogenic substrates have been used in the development of peroxidase DNAzyme sensors. Zhang et al. reported the use of thiamine in the detection of thrombin, with a detection limit of 1 pmol L^−1^ [15]. Other research groups focused on use of Amplex Red in DNAzyme systems for the detection of thrombin [16], glucose-oxidase activity [16], or lead ions [17]. With the use of this substrate, which oxidizes to the strongly fluorescent molecule resorufine, it was possible to develop methods for the analytes mentioned above with detection limits in the nanomolar range. However, new substrates are required for the development of sensitive biosensors.

In this paper we report a new fluorogenic substrate, *N*-methyl-4-hydrazino-7-nitrobenzofurazan (MNBDH), which can be oxidized to a fluorescing amino derivative (*N*-methyl-4-amino-7-nitrobenzofurazan (MNBDA)) in the presence of a DNAzyme with peroxidase-mimicking activity. MNBDH has several advantages over other fluorogenic substrates, which often require an alkaline pH and have a short excitation maximum wavelength. Use of MNBDH to develop a new, sensitive assay for DNA detection by a molecular-beacon probe is also presented.

## Materials and methods

### Reagents

All DNA oligonucleotides were purchased from Genomed S.A., Poland (HPLC purity) and were used without further purification. The sequences of the oligonucleotides able to form G-quadruplexes (G4 DNA) used in this study were:

**PS2.M**: 5′GTG GGT AGG GCG GGT TGG3′

**HT**: 5′ATT AGG GTT AGG GTT AGG GTT AGG G3′

**MB**: 5′*CCCT ACCC* **AGCC TTAA CTGT AGTA CTGG TGAA ATTG CTGC CATT** T *GGG TAGG G* CGG GTTG GG3′ (the loop region of MB is in bold, the duplex region of MB is in italic, and the G4-forming tract is underlined).

The target DNA (analyte) had a sequence complementary to a part of the MB probe: 5′AATG GCAG CAAT TTCA CCAG TACT ACAG TTAA GGC3′

The concentration of DNA was quantified by UV-Vis spectroscopy with the following extinction coefficients at 260 nm (mol^−1^ L cm^−1^): *A* = 15,400, *T* = 8700, *G* = 11,500, and *C* = 7400 [18]. All other chemicals were purchased from Sigma-Aldrich and were used without further purification. A hemin stock solution (10^−2^ mol L^−1^) was prepared in DMSO and stored in the dark at 30 °C for up to one month.

### Synthesis of *N*-methyl-4-hydrazino-7-nitrobenzofurazan

*N*-Methyl-4-hydrazino-7-nitrobenzofurazan (MNBDH) was synthesized based on a procedure by Büldt and Karst [19], modified as follows: to a solution of 4-chloro-7-nitrobenzofurazan (1 mmol) in ethanol (5 mL), methylhydrazine (1.23 mmol) in ethanol (1 mL) was added dropwise. The reaction mixture was heated under reflux, with stirring, for 20 min. Chloroform (5 mL) was added to the suspension, and the solution was stirred at 60 °C for 60 min. The product precipitated as red crystals after one day at room temperature. The precipitate was removed by filtration and washed with hexane. Recrystallization from a methanol–1,2-dichloroethane mixture resulted in a pure product with a 57 % yield. The purity of the product was confirmed by NMR and HPLC measurements. ^1^H NMR (400 MHz, DMSO-d_6_ + D_2_O): *δ* (ppm) = 3.88 (s, 3H, N–CH_3_), 6.6–6.81 (m, 1H, H_bʹ_), 8.46 (d, 1H, H_a_, J = 8.9 Hz).

### HPLC measurements

The HPLC chromatograms were recorded on a Waters chromatograph with photodiode (Model 2988) and fluorescence (Model 2475) detectors. The measurements were conducted before and after MNBDH oxidation with hydrogen peroxide, catalyzed by DNAzyme. The samples contained 10 μmol L^−1^ MNBDH, 1 μmol L^−1^ G4 DNA, 10 mmol L^−1^ Tris–HCl (pH = 8.0), 10 mmol L^−1^ KCl, 10 mmol L^−1^ NH_4_Cl, and 2 μmol L^−1^ hemin. The chromatogram of the solution after the peroxidase reaction was recorded 10 min after the addition of 5 mmol L^−1^ H_2_O_2_. All measurements were conducted using gradient conditions (10 % A, 90 % B → 60 % A, 40 % B) consisting of 60 % 10 mmol L^−1^ NaCl (solvent A) and 40 % acetonitrile (solvent B).

### DNAzyme-activity measurements

The characterization and optimization experiments were conducted using a Cary Eclipse Fluorescence Spectrometer (Agilent, Australia) and an M200 Microplate Reader (Tecan, Austria). The PS2.M oligonucleotide, in a solution containing 10 mmol L^−1^ Tris–HCl buffer (pH = 8.0), cations (100 mmol L^−1^ KCl, 400 mmol L^−1^ CH_3_COONH_4_), 2 μmol L^−1^ hemin, and 0.05 % Triton X-100, was heated for 5 min at 95 °C to ensure the denaturation and proper folding of the G-quadruplex. The mixture was cooled on ice for 15 min and then incubated for another 15 min at room temperature. After MNBDH addition (10 μmol L^−1^) the solution was incubated in the dark for another 30 min. The peroxidase reaction was initiated by the addition of H_2_O_2_ (1 mmol L^−1^). The peroxidation reaction product MNBDA had strong fluorescence at 540 nm with an excitation wavelength of 470 nm. For the molecular-beacon system, the probe (MB) solution was prepared and denatured as described above and, after 30 min incubation, a selected amount of analyte was added. The hybridization between MB and the analyte was performed for two hours. The next step involved the addition of the hemin required for DNAzyme creation. After the addition of MNBDH and H_2_O_2_, the fluorescence was monitored at 540 nm (*λ*_exc_ = 470 nm). The time-dependent fluorescence data obtained were analyzed by calculating the initial rate of reaction (*V*_init_, measured in counts s^−1^) from the increase in the fluorescence intensity (Δ*F* = *F* − *F*_0_, where *F* and *F*_0_ are the fluorescence intensity for the reaction catalyzed by DNAzyme and by hemin alone, respectively) within an initial 0–30 s time window. Error bars were calculated from *n* = 3 experiments.

### Melting profiles

The melting experiments were conducted on a Cary 300 UV-Vis Spectrophotometer (Agilent, Australia). The investigated systems contained 1 μmol L^−1^ G4 DNA, 10 mmol L^−1^ Tris–HCl (pH = 8.0), and 100 mmol L^−1^ KCl. The melting profiles were recorded in the range 10–90 °C with a rate of 1 ° min^−1^ and a data interval of 0.5 °.

### CD measurements

Before the CD measurements, sample solutions containing 1 μmol L^−1^ G4 DNA oligonucleotide, 10 mmol L^−1^ Tris–HCl (pH = 8.0), and adequate cation were denatured at 95 °C for 5 min and cooled on ice for 15 min. The CD spectra were measured with a 100 nm min^−1^ scan speed and a 1 nm bandwidth. All spectra were averaged from three scans.

## Results and discussion

**Fig. 1:**
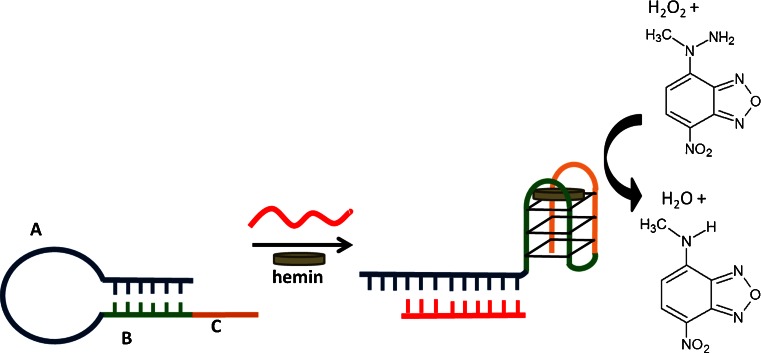
Scheme of the analytical approach for DNA detection based on a molecular-beacon probe that consists of the PS2.M sequence (domains *B* + *C*) and a domain *A* that is complementary to the analyte. The DNAzyme formed by the probe after analyte–DNA hybridization is able to catalyze MNBDH oxidation to MNBDA

The catalytic effectiveness of the peroxidase-mimicking DNAzyme system depends not only on the DNA sequence and, more specifically, the G-quadruplex topology but also on the reaction conditions [20]. Furthermore, each substrate may require a different optimum set of conditions [21]. The optimization is therefore the first and necessary step in the development of sensors using new peroxidase substrates. MNBDH was first used as a substrate for horseradish peroxidase by Meyer et al. [22]. This compound is nonfluorescent as a hydrazine derivative, but it can be oxidized by H_2_O_2_ in the presence of HRP or DNAzyme to *N*-methyl-4-amino-7-nitrobezofurazan (MNBDA), as shown in Fig. 1.

**Fig. 2:**
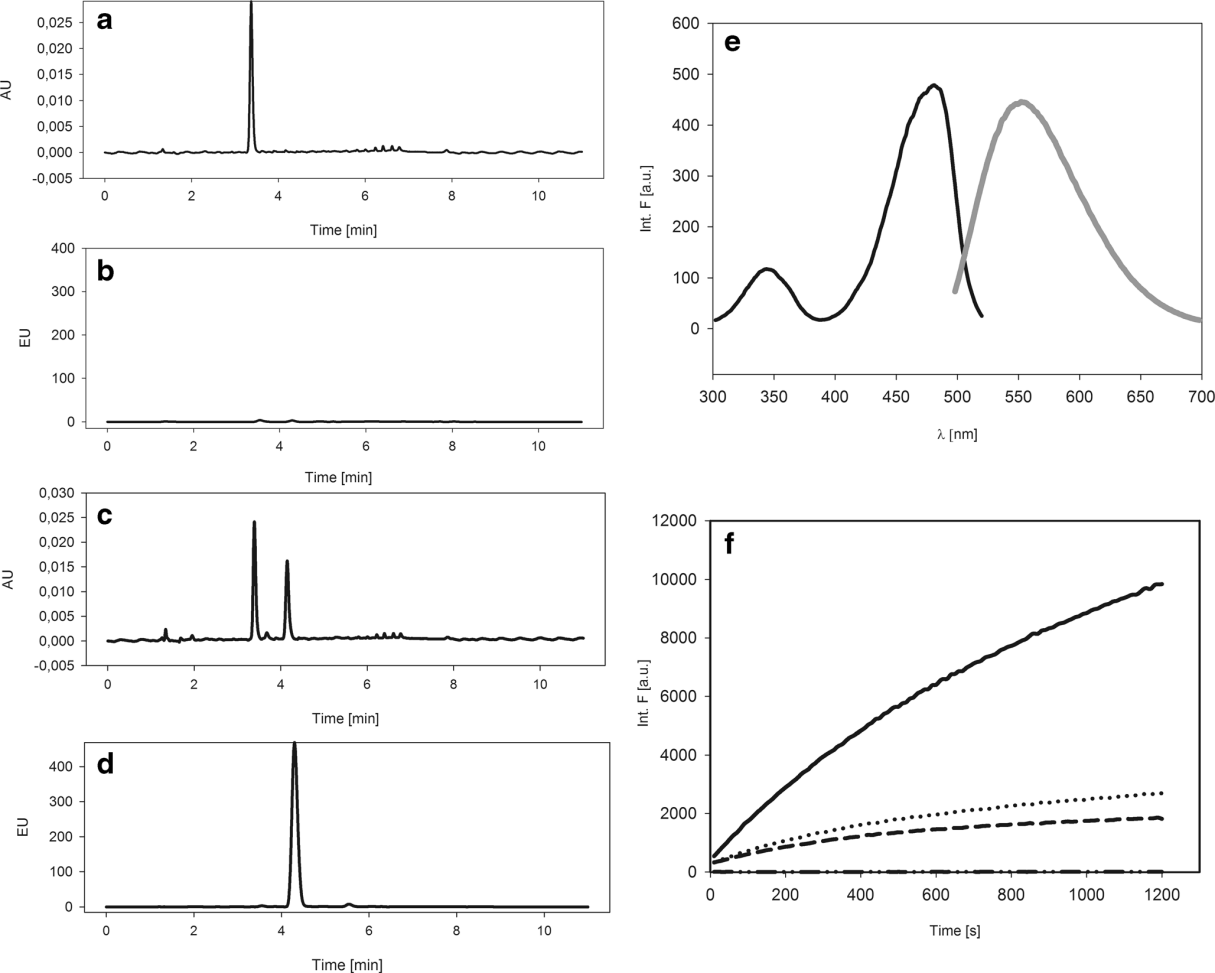
HPLC chromatogram of MNBDH with (**a**) colorimetric and (**b**) fluorescence detection. Chromatogram of MNBDH after the reaction catalyzed by DNAzyme, using the (**c**) colorimetric detector and (**d**) fluorescence detector. (**e**) Absorption (*black*) and fluorescence (*gray*) spectra of MNBDA: *λ*_ex_ = 450, *λ*_em_ = 540 nm, and 5-nm slits. (**f**) Dependence on time of the fluorescence intensity of the MNBDA for (*a*) 10 mmol L^−1^ Tris–HCl pH = 8.0, 100 mmol L^−1^ KCl (buffer A); (*b*) buffer A + 2 μmol L^−1^ hemin; (*c*) buffer A, 2 μmol L^−1^ hemin, 1 μmol L^−1^ HT; and (*d*) buffer A, 2 μmol L^−1^ hemin, 1 μmol L^−1^ PS2.M. All probes contained 100 μmol L^−1^ MNBDH, and the reaction was initiated by the injection of H_2_O_2_ into the final 100 μmol L^−1^ concentration. The kinetic traces were recorded for 20 min with 10 s intervals: *λ*_ex_ = 470, *λ*_em_ = 557 nm

As mentioned in the experimental section, the original procedure of MNBDH synthesis was modified to obtain a higher yield (57 %). The product of the synthesis was characterized by ^1^H NMR (experimental section) and UV-Vis spectroscopy, and the purity was confirmed by HPLC. Figures 2a, b show chromatograms for MNBDH only, and Figs. 2c, d show chromatograms for the reaction mixture after oxidation catalyzed by DNAzyme. The first chromatogram recorded with a diode-array detector (*λ* = 488 nm) yielded one peak at *R*_t_ = 3.4 min. This confirmed that the synthesized product is pure, can be used without further purification, and is not fluorescent (fluorescence detection). Next, the MNBDH was oxidized by H_2_O_2_ in the presence of DNAzyme for 10 min before HPLC separation, which was monitored by a diode array and a fluorescent detector (*λ*_ex_ = 480, *λ*_em_ = 556) and resulted in two peaks at 3.4 and 4.2 min with colorimetric detection and only one peak (*R*_t_ = 4.3) when a fluorescence detector was used. The intense peak in the chromatogram using a fluorescence detector corresponds to MNBDA formed by oxidation by the DNAzyme. The excitation and emission spectra of MNBDA are shown in Fig. 2e.

The optimization experiments were conducted on a microplate reader, which enabled parallel measurements of up to 96 samples. The optical system of this instrument is equipped with fixed slits (excitation at 9 nm, emission at 20 nm). For all optimization experiments, the kinetic changes of the fluorescence at 557 nm with excitation at 470 nm were recorded for 20 min with 10 s intervals. The excitation and emission wavelength were shifted from the maximum by use of a fixed 20 nm slit. Figure 2f gives the changes in the fluorescence of the system with reaction time for the buffer only (trace a); the hemin solution, which itself has some peroxidase activity (trace b); and the DNAzyme based on HT telomeric DNA (trace c) and on the PS2.M sequence (trace d). The intensity trace for the buffer reveals that the oxidation reaction does not occur without a catalyst. Hemin, as mentioned, had low peroxidase activity and can catalyze the oxidation of MNBDH to some extent, as indicated by a noticeable increase in the fluorescence signal (trace b). The DNAzyme based on the telomeric sequence (trace c) has activity only slightly higher than that for hemin alone. The modest activity of the DNAzyme based on the telomeric HT sequence can be explained by its weak binding affinity to hemin [23]. The highest activity is produced by the system based on the PS2.M sequence, which is regarded as a model peroxidase-mimicking DNAzyme. This experiment confirmed that the peroxidase-mimicking DNAzyme successfully catalyzed the oxidation of MNBDH and that this substrate can be used for the development of sensors based on the deoxyribozyme approach.

**Fig. 3:**
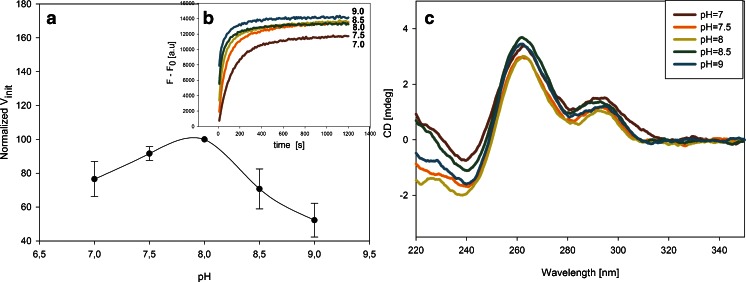
Effect of pH on the MNBDH oxidation catalyzed by DNAzyme based on the PS2.M sequence. (**a**) Dependence of the normalized initial rate on the pH of the reaction solution. (**b**) Kinetic profiles (fluorescence changes) of the MNBDA generated in the peroxidase reaction at selected pH values of 10 mmol L^−1^ Tris–HCl buffer (1 μmol L^−1^ G4 DNA, 2 μmol L^−1^ hemin, 100 mmol L^−1^ KCl, 100 μmol L^−1^ MNBDH, and 15 mmol L^−1^ H_2_O_2_). (**c**) CD spectra of the PS2.M quadruplex at different pH values (1 μmol L^−1^ DNA, 10 mmol L^−1^ Tris–HCl, and 100 mmol L^−1^ KCl)

The first factor that we considered in the evaluation of the reaction conditions was the pH of the buffer. Travascio et al., in their first paper on a peroxidase-mimicking DNAzyme, reported that the highest catalytic activity for their system with ABTS as a substrate was observed in the pH range 7–9, with the maximum at approximately 8.5. With that in mind, we measured the catalytic activity of DNAzyme based on the PS2.M sequence in the oxidation of MNBDH in the same pH range of 7–9. The fluorescence intensity changes with reaction time for different pH values are shown in Fig. 3. However, the fluorescence intensity is not a convenient variable for the characterization of kinetic data; the initial reaction rate better reflects the enzyme efficiency. The initial reaction rate (*V*_init_) can be easily determined from the fluorescence intensity vs. time. As shown in Fig. 3, the highest *V*_init_ for MNBDH oxidation with H_2_O_2_ catalyzed by DNAzyme is observed at pH 8.0, and all further experiments were conducted at this buffer pH. To verify whether changes in the activity of the system with pH were connected with the DNAzyme structural alteration, circular-dichroism (CD) experiments were conducted at different pH values. In the presence of potassium ions, the PS2.M oligonucleotide forms a hybrid G-quadruplex or a mixture of parallel and antiparallel structures [24]. On the CD spectra, this is represented by two positive bands at 260 and 295 nm and a negative band at 240 nm. As shown in Fig. 3b, no substantial changes are observed in the spectra of the PS2.M quadruplex at different pH values, which can be regarded as a proof that the G-quadruplex does not change its structure. The CD band at 260 nm is significantly more intense than that at 295 nm, indicating that a quadruplex with a parallel arrangement dominates in this mixture.

**Fig. 4:**
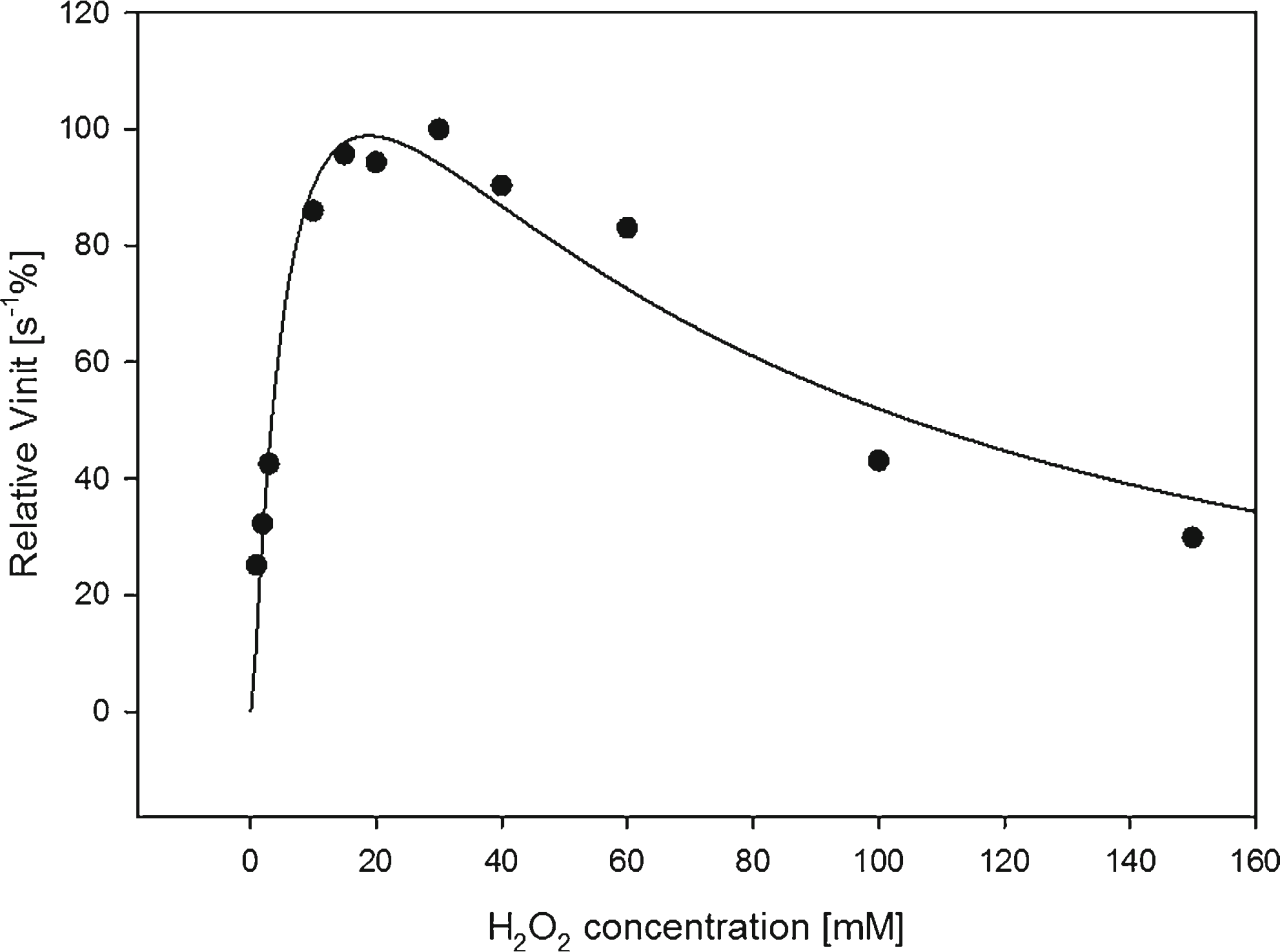
Effect of hydrogen peroxide on *V*_init_ of the DNAzyme-catalyzed oxidation of MNBDH. Conditions: 1 μmol L^−1^ PS2.M, 2 μmol L^−1^ hemin, 10 mmol L^−1^ Tris–HCl pH = 8.0, 100 μmol L^−1^ MNBDH, 100 mmol L^−1^ KCl, and 0.05 % Triton X-100. The remaining conditions are as in Fig. 3

In the next step, the effect of the H_2_O_2_ concentration on the catalyzed reaction was examined. The peroxidase reaction rate was found to depend only on the H_2_O_2_ concentration, because the binding of hydrogen peroxide by hemin is the limiting step of this reaction [25]. Thus, with an increase in the concentration of hydrogen peroxide, the rate of reaction should also increase. However, H_2_O_2_ is suspected to also have other effects on the system because of its oxidative potential, which can damage the DNAzyme. Because of these two opposing effects, it is essential to determine the optimum concentration of hydrogen peroxide. We examined the effect of the H_2_O_2_ concentration on the DNAzyme activity in the range 10 μmol L^−1^–150 mmol L^−1^ (Fig. 4). With increasing concentration of hydrogen peroxide, the *V*_init_ also initially increases, reaching a maximum at an H_2_O_2_ concentration of 20–30 mmol L^−1^. After this point, the reaction rate drops instantly. As mentioned earlier, this reduction may be connected with the DNAzyme damage by hydrogen peroxide. The optimum concentration ratio of H_2_O_2_ to MNBDH was calculated to be approximately 300.

**Fig. 5:**
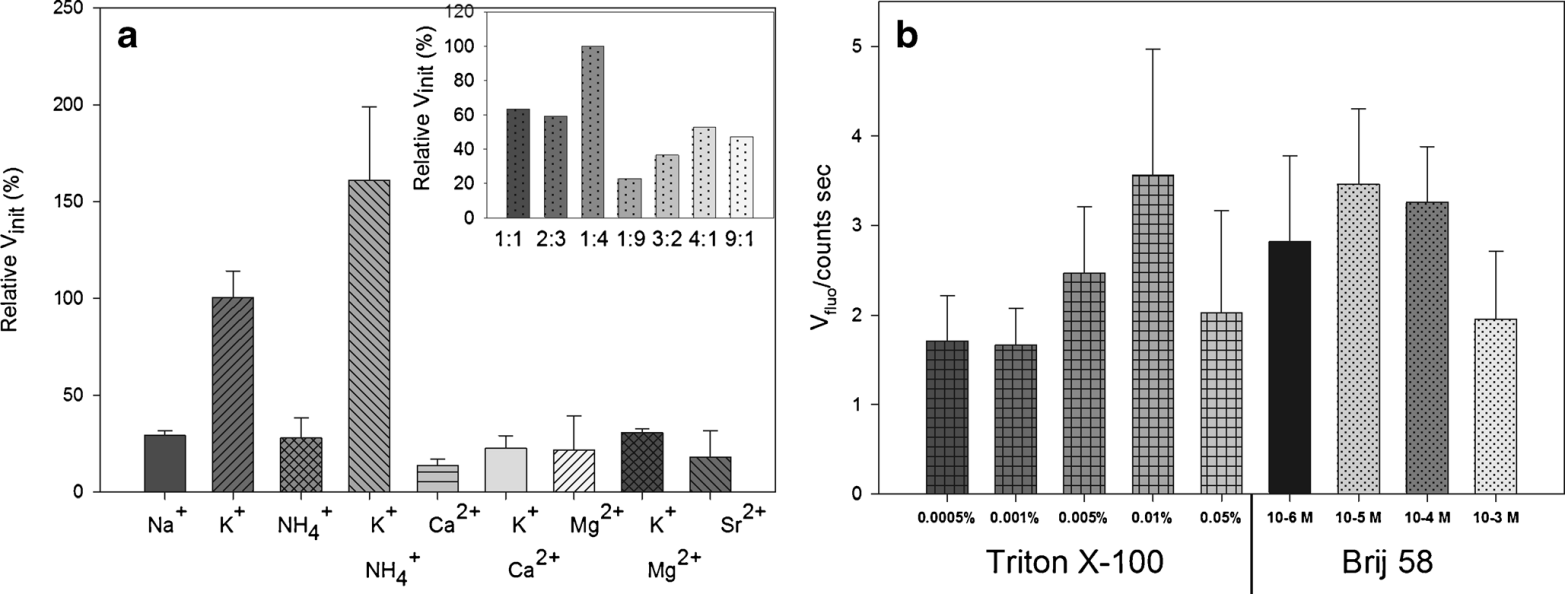
The effect of the type and concentration of cations (**a**) and surfactants (**b**) on the initial rate of the MNBDH oxidation catalyzed by the peroxidase-mimicking DNAzyme. (**a**) Relative initial velocity of the reaction in the samples containing different cations. Insert: the effect of the concentration ratio of K^+^/NH_4_^+^ on the initial velocity. (**b**) Initial reaction rates for the systems containing the surfactants Triton X-100 and Brij 58 at the selected concentrations. All conditions are as in Fig. 3

One of the most important factors that affects the DNAzyme activity is the type of cation involved in the quadruplex formation and its concentration. The G-quadruplex structure is stabilized by three main forces. First, there are Hoogsteen-type hydrogen bonds between the guanine residues. Second, hydrophobic interactions operate between the planar guanine rings, which enable the stacking of bases on top of each other. Finally, the electrostatic interaction between the phosphoric-acid residues and the metal cations can reduce the repulsive forces between the anionic phosphates. Moreover, depending on the ionic radius, a cation can penetrate the channel created in the middle of the G4 structure, in which it may undergo coordination by guanine carbonyl groups. A guanine-rich oligonucleotide can form different topologies of the G-quadruplex depending on the nature (ionic radius) of the cation. This, in turn, has an effect on the G4–hemin binding properties. The parallel topology has been proved to be the structure with the highest affinity for binding hemin because of the accessible external G-quartets, which enable the end-stacking mode of hemin binding [23]. In contrast, antiparallel topologies have protruding loops in external G-quartets, which create spatial barriers for hemin binding. According to literature reports, in the presence of sodium cations the PS2.M oligonucleotide forms an antiparallel topology, whereas potassium ions induce the formation of a hybrid structure or a mixture of parallel and antiparallel structures [24]. Most papers suggest that potassium cations are necessary to ensure proper G-quadruplex folding and to preserve the DNAzyme activity [26]. Some reports suggest that the presence of other cations with potassium can positively alter the DNAzyme efficiency [21]. Therefore, we investigated how the type of cation affected the MNBDH oxidation catalyzed by DNAzyme based on the PS2.M sequence. The results are presented in Fig. 5a. The initial velocities for particular systems were calculated in relation to the activity of a sample containing an excess of potassium ions (100 %). We examined the effect of mono (K^+^, Na^+^, NH_4_^+^) and divalent (Mg^2+^, Ca^2+^, Sr^2+^) cations. The addition of a second cation with potassium was also evaluated. The highest initial rate was observed for the system containing potassium and ammonium cations together (ca. 150 %). Therefore, in the next step, we examined the effect of the ratio of the K^+^ to NH_4_^+^ concentrations (Fig. 5a insert), and 100 mmol L^−1^ K^+^ with 400 mmol L^−1^ NH_4_^+^ was the most effective composition for this reaction. A mixture of K^+^ and NH_4_^+^ cations at this ratio was therefore used in further experiments.

The final optimization experiments focused on the type and concentration of surfactant. We investigated two surfactants: Triton X-100, which is commonly used in all peroxidase-DNAzyme assays, and Brij 58, which we reported to be a good alternative to Triton X-100 [19]. For Triton X-100, the best results were obtained at a surfactant concentration of 0.005 % (*ca*. 8 × 10^−5^ mol L^−1^) (Fig. 5b), whereas the highest initial rate for Brij 58 was observed at a concentration of 1 × 10^−5^ mol L^−1^. The CMC values for Triton X-100 and Brij 58 are approximately 2.4 × 10^−4^ and 0.8 × 10^−4^ mol L^−1^, respectively. This implies that, for the DNAzymes, the preferential concentration of the surfactant is below its CMC value, meaning that micelles are not yet formed. The results obtained with Brij 58 as the surfactant were slightly better than those for the system with Triton X-100. For further experiments, we recommend the use of Brij 58 as the surfactant in DNAzyme assays.

The optimized oxidation of MNBDH by peroxidase DNAzyme can be used in the development of sensitive assays. Here, we present the procedure for target-DNA-sequence detection using a molecular-beacon probe. A depiction of the system design is presented in Fig. 1. The molecular-beacon probe consists of three elements. Part A contains two segments, one that is complementary to the analyte and a second that is complementary to domain B (both form the duplex portion of the MB structure). Domains B and C together represent the PS2.M sequence. The G-quadruplex structure cannot be formed because part of the PS2.M sequence is involved in the duplex formation. When the analyte is present, it can hybridize with the loop of the MB structure and thereby opens the molecular beacon. The PS2.M sequence, which is thus released, can form a G-quadruplex structure and, after hemin binding, forms a DNAzyme able to catalyze the peroxidase reaction. There is an alternative blunt-end structure for the MB used in this study that contains a T-T mismatch in the duplex region. This can affect the stability of the MB structure in the presence of Hg^2+^ ions [27]. This is not, however, significant because of the low concentration of this metal in biological samples. The MB system is a very popular design in the development of bioassays, and has also been used in combination with a DNAzyme with peroxidase activity [14]. However, the reported detection limit of 0.2 μmol L^−1^ obtained with ABTS as a substrate can be significantly improved by the use of a fluorogenic substrate, for example MNBDH.

**Fig. 6:**
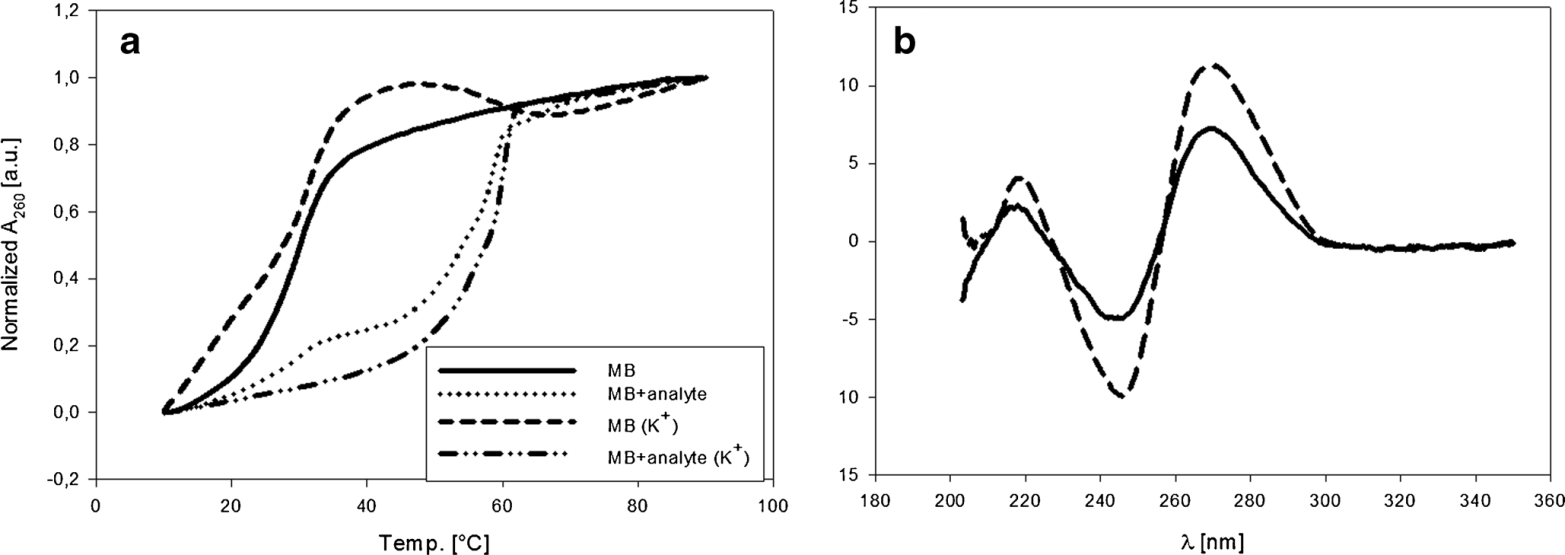
Characterization of the MB–analyte system. (**a**) Melting profiles of the MB systems in the absence and presence of 100 mmol L^−1^ potassium. The *solid* line represents MB alone, the *dotted* line represents MB with the analyte, the *dashed* line represents MB in the presence of potassium cations, and the *dash*-*dot* line represents MB as an analyte in the presence of K^+^. Conditions: 1 μmol L^−1^ DNA (MB or analyte), and 10 mmol L^−1^ Tris–HCl pH = 8.0. (**b**) CD spectra of MB (*black*) and MB + analyte (*dashed*): 1 μmol L^−1^ DNA, 10 mmol L^−1^ Tris–HCl pH = 8.0, and 100 mmol L^−1^ KCl

To characterize the MB construct and to ensure that the entire system worked properly, we conducted melting and CD experiments. Melting profiles were obtained by measuring the absorbance changes in relation to temperature. Figure 6a represents the system without cations present, in which a G-quadruplex cannot be formed. The molecular beacon has a melting temperature (*Tm*) (which indicates half the DNA oligonucleotide to be melted) of approximately 28 °C, which characterizes the double-stranded stem of the MB. When the analyte is present, two waves can be distinguished on the melting curve. The first is at a *Tm* of 27 °C, and the second is at a *Tm* of approximately 56 °C. The first melting step corresponds to a fraction of the molecular beacon which is in the native form (it is a similar *Tm* value to that of the system without the analyte). The second melting step corresponds to the denaturation of the MB–analyte duplex. When potassium cations are present, the MB alone has *Tm* = 31 °C (Fig. 6a). The addition of analyte causes a shift of the melting temperature to 59 °C, but only a single melting step is observed. The G-quadruplex probably has a similar melting temperature to that of the MB–analyte duplex. The values of the melting temperatures for all tested systems are collected in Table 1. These results confirmed that the MB and analyte hybridized and formed a more stable duplex than that present in the stem of the MB structure. Unfortunately, the results obtained at 295 nm, the wavelength chosen as the best to observe formation of the G4 structure, seemed not to be suitable for *Tm* determination. This is most probably because of the complexity of the system, resulting in the overlapping of spectral changes associated with the simultaneous melting of the double-stranded DNA and the G-quadruplex. To verify the structural changes of MB, CD experiments were conducted (Fig. 6b). Both for MB alone and with addition of the analyte, the positive bands at 270 nm and negative bands at 245 nm are present. The addition of the analyte causes an increase in the band intensities. Depending on the topology, the G-quadruplexes have characteristic CD bands: there is a positive band at 260 nm and a negative band at 240 nm for parallel G4, whereas the antiparallel structure has a positive band at 290 nm and negative band at 260 nm. A common feature of the G-quadruplexes is an additional characteristic peak at 210 nm. For duplex DNA, a positive band at 260–280 nm and a negative band at 245 nm are observed [28]. It is difficult to determine definitely whether the observed CD bands (Fig. 6b) are connected with the G-quadruplex or the duplex structure. Most probably, each recorded spectrum is a superposition of the bands from these two DNA structures. Although the melting profiles and CD spectra did not provide clear evidence of G-quadruplex formation, the duplex formation after analyte addition (Fig. 6a) enables one to conclude that the PS2.M domain is unbound and is free to form the G-quadruplex structure.

**Table 1:** Melting temperatures (*Tm*) for all melting systems

	*Tm* MB (°C)	*Tm* MB + analyte (°C)	
Without metal ions	28.8 ± 1	*Tm*_1_	*Tm*_2_
		27.0 ± 0.1	56.7 ± 0.2
K^+^	30.9 ± 0.5	59.3 ± 0.6	

**Fig. 7:**
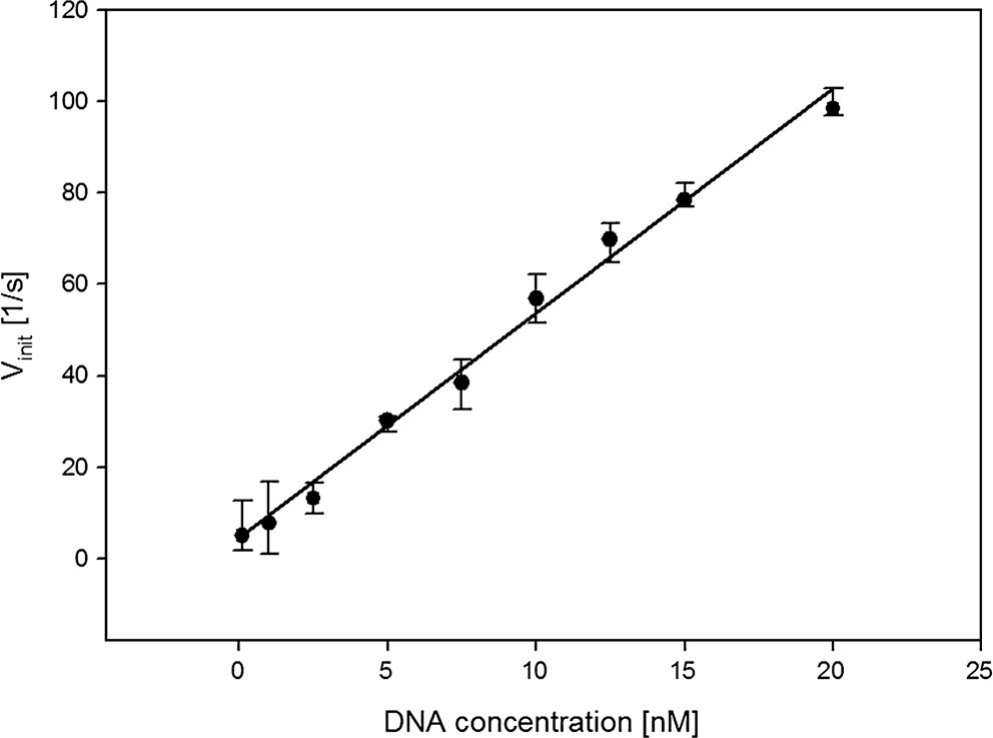
Analytical performance of the MB-probe-based DNAzyme fluorimetric DNA assay. Calibration curve for the DNA-analyte determination using the DNAzyme-based MB assay with the fluorogenic MNBDH substrate. Conditions: 20 nmol L^−1^ MB, 40 nmol L^−1^ hemin, 5 μmol L^−1^ MNBDH, and 1.5 mmol L^−1^ H_2_O_2_

The optimized and characterized system was next used to develop an assay for DNA detection. The experiments were conducted in the 0.1–20 nmol L^−1^ concentration range of the target DNA by the procedure described in the experimental section. The calibration curve based on the dependence of the initial rate on the analyte concentration is linear (Fig. 7) and confirms that fluorogenic MNBDH can be successfully incorporated into assays based on peroxidase-mimicking DNAzymes. The detection limit of the assay, calculated from the standard deviation of the blank, corresponds to a concentration of the analyte (DNA strand) of 1 nmol L^−1^.

## Conclusions

This study introduced a new fluorogenic substrate for assays based on peroxidase-mimicking DNAzymes. The oxidation reaction of *N*-methyl-4-hydrazino-7-nitrobenzofurazan by H_2_O_2_ was optimized to obtain a high fluorescence signal. It has been revealed that this system has the highest activity at pH 8. The ratio of c_H2O2_/c_MNBDH_ ~ 300 produced the best results. In the optimization experiments, the best types of cation (K^+^ + NH_4_^+^) and surfactant (Brij 58) were also selected. The optimized reaction was then used to detect a DNA sequence by use of a molecular-beacon probe (MB). The MB–analyte system was characterized by recording the circular-dichroism spectra and melting profiles. The experiment with different concentrations of the analyte proved that this system can be successfully used in assays for DNA detection. The calibration curve was linear in the range of 1–20 nmol L^−1^ of analyte. The detection limit for the setup was estimated to be 1 nmol L^−1^. The results proved that MNBDH as a fluorogenic substrate and peroxidase DNAzyme as a biocatalyst form a sensitive system for bioassay development. Further application studies are in progress.
